# Malignancy risk estimation of pulmonary nodules in screening CTs: Comparison between a computer model and human observers

**DOI:** 10.1371/journal.pone.0185032

**Published:** 2017-11-09

**Authors:** Sarah J. van Riel, Francesco Ciompi, Mathilde M. Winkler Wille, Asger Dirksen, Stephen Lam, Ernst Th. Scholten, Santiago E. Rossi, Nicola Sverzellati, Matiullah Naqibullah, Rianne Wittenberg, Marieke C. Hovinga-de Boer, Miranda Snoeren, Liesbeth Peters-Bax, Onno Mets, Monique Brink, Mathias Prokop, Cornelia Schaefer-Prokop, Bram van Ginneken

**Affiliations:** 1 Department of Radiology and Nuclear Medicine, Radboud University Medical Center, Nijmegen, The Netherlands; 2 Department of Diagnostic Imaging, Section of Radiology, Nordsjællands Hospital, Hillerød, Denmark; 3 Department of Pulmonology, Gentofte Hospital, University of Copenhagen, Hellerup, Denmark; 4 Department of Integrative Oncology, British Columbia Cancer Agency, Vancouver, Canada; 5 Department of Radiology, Centro de Diagnostico Dr Enrique Rossi, Buenos Aires, Argentina; 6 Department of Clinical Sciences, Division of Radiology, University Hospital of Parma, Parma, Italy; 7 Department of Radiology, Vrije Universiteit Medisch Centrum, Amsterdam, the Netherlands; 8 Department of Radiology, Meander Medical Center, Amersfoort, the Netherlands; 9 Department of Radiology, UMC Utrecht, Utrecht, the Netherlands; Peking University People's Hospital, CHINA

## Abstract

**Purpose:**

To compare human observers to a mathematically derived computer model for differentiation between malignant and benign pulmonary nodules detected on baseline screening computed tomography (CT) scans.

**Methods:**

A case-cohort study design was chosen. The study group consisted of 300 chest CT scans from the Danish Lung Cancer Screening Trial (DLCST). It included all scans with proven malignancies (n = 62) and two subsets of randomly selected baseline scans with benign nodules of all sizes (n = 120) and matched in size to the cancers, respectively (n = 118). Eleven observers and the computer model (PanCan) assigned a malignancy probability score to each nodule. Performances were expressed by area under the ROC curve (AUC). Performance differences were tested using the Dorfman, Berbaum and Metz method. Seven observers assessed morphological nodule characteristics using a predefined list. Differences in morphological features between malignant and size-matched benign nodules were analyzed using chi-square analysis with Bonferroni correction. A significant difference was defined at p < 0.004.

**Results:**

Performances of the model and observers were equivalent (AUC 0.932 versus 0.910, p = 0.184) for risk-assessment of malignant and benign nodules of all sizes. However, human readers performed superior to the computer model for differentiating malignant nodules from size-matched benign nodules (AUC 0.819 versus 0.706, p < 0.001). Large variations between observers were seen for ROC areas and ranges of risk scores. Morphological findings indicative of malignancy referred to border characteristics (spiculation, p < 0.001) and perinodular architectural deformation (distortion of surrounding lung parenchyma architecture, p < 0.001; pleural retraction, p = 0.002).

**Conclusions:**

Computer model and human observers perform equivalent for differentiating malignant from randomly selected benign nodules, confirming the high potential of computer models for nodule risk estimation in population based screening studies. However, computer models highly rely on size as discriminator. Incorporation of other morphological criteria used by human observers to superiorly discriminate size-matched malignant from benign nodules, will further improve computer performance.

## Introduction

Lung cancer is the deadliest cancer in both men and women [1] because the disease is usually diagnosed at an advanced stage. The National Lung Screening Trial (NLST) demonstrated a decrease in lung cancer-specific mortality of 20% [2], which was the major driving power leading to a recommendation of low-dose computed tomography (CT) lung screening for eligible subjects by several organizations including the U.S Preventative Services Task Force (USPSTF) [3,4]. Lung cancer screening programs are now being implemented in the U.S.

Low-dose CT studies of the lung—obtained within a lung cancer screening program—are characterized by the discrepancy between the high prevalence of pulmonary nodules and the relatively low incidence of actual lung cancers. In the first CT round of the 24,715 subjects in the CT arm of the NLST a total of 6,786 nodules had been annotated, however, only 168 subjects were eventually diagnosed with a pulmonary malignancy within the 3 years screening period or the follow-up of 6.5 years, illustrating the high prevalence of nodules in screening CTs in general and the magnitude of the diagnostic and organizational challenge [5]. Accuracy and efficiency of screening programs will therefore largely depend on the ability to correctly differentiate malignant from benign nodules. A prospective estimation of which nodules are of high risk to represent or develop into a malignancy, and therefore requiring more close follow-up or intense diagnostic work-up as opposed to nodules that are of very low risk, is of crucial importance to make screening programs efficient for a number of reasons including burden of radiation dose, psychological load of the screening subjects and financial expenses. This problem has been addressed by the management guidelines published by the American College of Radiologists (ACR), called Lung-RADS. Nodules with benign appearance and very low risk (categories 1 and 2) are differentiated from equivocal or suspicious nodules (categories 3, 4A and 4B) [6]. Lung-RADS and similar recommendations [7–9] have in common that they use nodule type, nodule size and growth rate to select nodules that are malignant or at risk to develop into a malignancy. A number of other risk prediction models rather focus on the selection of individuals being at risk for developing a lung malignancy [10–13] or estimate the malignancy probability using both, clinical factors and nodule characteristics [7, 14–15].

The computer model that was evaluated in the following study also uses both a number of nodule characteristics but also non-nodular CT imaging findings and further on considers a number of subject related demographic factors [16]. This model is thus far the only published malignancy risk prediction model that was mathematically modeled based on the findings of an actual lung cancer screening trial [17]. Whereas other models use fixed thresholds for nodule size this model applies hazard ratios for various parameters that have been derived from the Pan-Canadian Early Detection of Lung Cancer Study (therefore the name PanCan model). The model was externally validated using the British Columbia Cancer Agency (BCCA) cohort and demonstrated a good performance with an area under the receiver-operator-characteristics curve (AUC) of > 0.900 to discriminate malignant from benign nodules [16]. An external second evaluation study used the cohort of the Danish Lung Cancer Screening Trial (DLCST) and found an AUC of 0.834 [18]. The difference in results between the two external validation studies can be explained by the fact that the performance of such risk prediction models are considerably influenced by the type of nodule annotation protocol (e.g., how many small, most likely benign nodules were included), the prevalence of malignancy and the underlying screening population.

In contrast to mathematical risk prediction models, radiologists use a rather complex system of visually accessible morphological information when determining the malignancy risk of pulmonary nodules, such as internal nodular and external perinodular characteristics of the lung parenchyma in addition to nodule diameter, upper lobe location and nodule type. No studies thus far have investigated the difference in performance between the PanCan model and human observers. The purpose of our study was to compare the PanCan model with human observer performance for the prediction of malignancy risk of screen-detected nodules.

## Methods

### Materials

For this retrospective study, we used anonymized CT scans from the Danish Lung Cancer Screening Trial [19]. Approval by the Ethics Committee of Copenhagen County as well as informed consent of all participants were available. In the DLCST, two experienced chest radiologists had separately annotated nodules (≥ 3 mm) in CT scans with respect to size and location, based on visual assessment and manual diameter measurements [19]. Follow-up information with respect to presence of histologically proven malignancies was available for a follow up of 9 years. Malignancies had a medium diameter of 15 mm (range 4 to 93 mm) on the first scans they had been annotated.

For the observer study a case-cohort study design was used. A study-group of 300 participants was selected from the complete screening data set under the following conditions: The study group included all 60 participants with at least one malignant nodule that had been found in the complete DLCST (group 1), 120 participants with at least one benign nodule randomly selected from the whole screening dataset (group 2), and 120 participants also randomly selected from the whole screening dataset but under the condition that they showed at least one benign nodule with a diameter in the range of 3 to 16 mm with a preference for lesions larger than 10 mm (group 3).

Group 1 consisted of the CT scans on which the malignant nodules had been annotated first. In participants with multiple malignant lesions, one malignant nodule was randomly selected. Group 2 consisted of baseline CT scans with benign nodules of all size ranges being reflective of lesions seen in a screening cohort. Group 3 consisted of baseline CT scans with benign nodules with a medium size larger than seen in the whole cohort but matching in size more closely to the malignant nodules.

Two size-matched nodules that had been classified as benign during the screening rounds developed into malignancies during the follow-up period of 9 years, their status was therefore changed accordingly resulting in a final group of 62 malignancies, 120 random benign and 118 size-matched benign nodules.

### CT acquisition

All CT scans were performed using volumetric acquisition (16 rows Philips Mx 8000, Philips Medical Systems, Eindhoven, The Netherlands) and a low-dose protocol (120 kV, 40 mAs) with a section collimation of 16 x 0.75 mm, pitch 1.5 and rotation time 0.5 s. The scans were obtained after full inspiration and without the use of contrast. Images were reconstructed with thin (1 mm) sections and a sharp filter algorithm (kernel D) [19].

### Readers and image viewing

Eleven observers assessed the nodules in random order, using a computer vision tool (CIRRUS Observer, Diagnostic Image Analysis Group, RadboudUMC, Nijmegen, The Netherlands) that allowed for interactive viewing of high quality CT sections in all 3 projections (axial, coronal and sagittal). The CT data were preloaded and displayed using a zoomed view of each nodule to shorten the search process, however, observers could scroll and review the complete CT if warranted. They were instructed to give a malignancy probability score to each nodule on a scale of 0 to 100, where a score of 0 would indicate that all nodules that looked like this particular nodule would be benign according to the observer and a score of 100 would indicate that all similar looking nodules would be malignant according to the observer. The observers assessed the malignancy score on the basis of visual analysis of nodule morphology. Judgment was left to the readers' discretion and no specific criteria were given being suggestive for malignancy.

Subsequently, the observers scored the presence of certain morphological features for each nodule according to a predefined list. This list included the following items: single bubble, multiple bubbles, airbronchogram, bulla with thickened wall, spiculation, lobulation, ill-defined border, well-defined border, demarcation by interlobular septa, attachment to a vessel, pleura or fissure, retraction of pleura or fissure, and distortion of surrounding lung architecture. Scoring the morphology features was not mandatory, however, if they decided to score nodule morphology, they were urged to do so for all nodules. Prior to scoring the actual study group, 15 example cases were presented to the observers, in order to get familiar with the software, nodule scoring method and the morphological features.

The observer group consisted of four board certified radiologists with > 10 years of experience in reading chest CTs and/or intense research training with respect to interpreting screen-detected nodules, five radiology residents and two pulmonologists from eight institutions in five countries. The first group of the four board certified radiologists formed the group of observers of higher experience as opposed to the remaining seven observers.

### PanCan computer model

We used the full model (2b), which determines the malignancy probability score based on the parameters sex, age, family history of lung cancer, emphysema, nodule size, nodule type, nodule location, nodule count and spiculation. Additionally, performance of the parsimonious model (1b) was assessed, which includes a limited number of input parameters such as sex, nodule size, nodule location and spiculation [16], thus leaving out age, family history, emphysema, nodule type and nodule count. Family history of lung cancer pertained to parents or siblings. Presence of emphysema was dichotomous and not corresponding to the degree of emphysema. Nodule size was measured as the longest diameter. Nodule count pertained to the number of additional nodules in the scan. Spiculation was defined as reticular markings of tissue density centered along the border of the nodule. Nodule type, emphysema, spiculation, and complete calcification were scored by an experienced radiologist (E.S.) [16] as this information was not available from the DLCST database. The remaining parameters such as sex, age, family history, nodule size, location and count were available from the DLCST database. Completely calcified nodules and perifissural nodules (PFN) were given a malignancy probability score of 0 according to the model. The latter refer to smoothly defined nodules smaller than 5 mm in size that are localized on or very close to the interlobar fissure and correspond to benign intrapulmonary lymph nodes [20].

### Statistical analysis

To compare the performance of the PanCan model with each observers' performance, a multi-reader multi-case (MRMC) receiver operating characteristic (ROC) analysis was applied using the PROPROC method [21–23]. Areas under ROC curve (AUCs) of observers and the full PanCan model were compared twice: a) considering the scores for all malignant and the set of randomly selected benign nodules, and separately when b) considering the scores for all malignant and the set of size-matched benign nodules. Performance differences were tested using the Dorfman, Berbaum and Metz method (DBM-MRMC package, version 2.33, http://perception.radiology.uiowa.edu), which accounts for case, reader, and treatment variance.

Differences in dataset characteristics were compared using chi-square for categorical parameters and unpaired t-test analyses for continuous variables. A significant difference was defined at p < 0.05. Morphological nodule features were considered to be present in the final data analysis when the majority of the observers had scored its presence positively. Differences in morphological features between malignant and size-matched benign nodules were analyzed using chi-square analysis. With Bonferroni correction, a significant difference was defined at p < 0.004. Analyses were performed using SPSS 22.0 (SPSS Inc., Chicago, IL, USA).

## Results

Characteristics of subjects, subgroups and nodules as annotated in the screening database are summarized in Table 1. The time between the CT scan used in this study and the date of diagnosis was on average 20.5 months (range 0.3–86 months); the follow-up time for the benign nodules was on average 121.1 months (range 109.1–126.7 months).

**Table 1:** Characteristics of the nodule groups.

Parameters PanCan model	Malignant nodules*	Random benign nodules	Size-matched benign nodules	Total	P value Size-matched / random †
**Number**	62	120	118	300	
**Age in years**	62 (52–75)	58 (50–68)	58 (50–69)	59 (50–75)	0.927 / 0.703
**Sex: Male; Female**	33 (53%); 29 (47%)	59 (49%); 61 (51%)	60 (51%); 58 (49%)	152 (51%); 148 (49%)	0.875 / 0.598
**Family history of lung cancer**	16 (26%)	21 (18%)	28 (24%)	65 (22%)	0.245 / 0.624
**Emphysema**	46 (74%)	62 (52%)	87 (74%)	195 (65%)	1.000 / **0.005**
**Nodule size in mm**	15 (4–93); Median: 12	6 (3–16); Median: 5	12 (3–90); Median: 9	10 (3–93); Median: 7	**0.003 / <0.001**
**Nodule Type: Solid; Part-solid; Non-solid; Perifissural**	45 (73%); 10 (16%); 7 (11%); 0 (0%)	107 (89%); 1 (1%); 3 (2.5%); 9 (7.5%)	84 (71%); 11 (9%); 16 (14%); 7 (6%)	236 (79%); 22 (7%); 26 (9%); 16 (5%)	0.137 / **<0.001**
**Nodule Count**	1.3 (1–5)	1.85 (1–6)	1.6 (1–4)	1.6 (1–6)	**0.042 / 0.004**
**Nodule Location: Upper Lobe**	39 (63%)	50 (42%)	64 (54%)	153 (51%)	0.273 / **0.006**
**Spiculation**	22 (35%)	3 (3%)	7 (6%)	32 (11%)	**<0.001 / <0.001**
**Calcified**	0 (0%)	24 (20%)	16 (14%)	40 (13%)	**0.001 / <0.001**

* Percentages or ranges are in parentheses.

^†^ P-value for malignant and size-matched benign nodules / malignant and randomly selected benign nodules. A p-value < 0.05 indicates significance of difference. Significant differences are indicated in bold. Note that malignant nodules are more often spiculated, have a higher nodule count, and are on average 3 mm larger than the size-matched benign nodules. Malignant nodules are more often part-solid nodules and located in the upper lobes compared to the benign nodule groups, while randomly selected benign nodules are mostly small solid nodules.

Nodule size, the presence of spiculation, the presence of nodule calcification, and the number of nodules per scan (nodule count) were statistically significantly different between malignancies and size-matched benign nodules (p = 0.003, p < 0.001, p = 0.001, and p = 0.042, respectively) as well as between malignancies and randomly selected benign nodules (p < 0.001, p < 0.001, p < 0.001, and p = 0.004, respectively). Additionally, nodule type (p < 0.001), nodule location in the upper lobe (p = 0.006), and the presence of emphysema (p = 0.005) were statistically significantly different between malignancies and randomly selected benign nodules (Table 1).

### Performance of observers versus the computer model

For discriminating randomly selected benign nodules (group 2) from malignant nodules (group 1), the PanCan model 2b had an AUC of 0.932 and the human observers had an average AUC of 0.910 (range 0.860–0.950); the difference did not achieve significance (p = 0.184). The four board certified radiologists yielded an averaged AUC of 0.919, whereas the five radiology residents and two pulmonologists had an AUC of 0.905. Both were not significantly different from the computer model (p = 0.366 and p = 0.117, respectively).

Human observers achieved an average AUC of 0.819 (range 0.771–0.881) for differentiating size-matched benign nodules (group 3) from malignancies (group 1), while the computer model achieved an AUC of 0.706. The difference between the mean performance of all observers and the computer model was statistically significant (p < 0.001). The differences between each individual observer and the computer model were statistically significant for 10 of the 11 observers (range from p < 0.001 to p = 0.022). The board certified radiologists yielded an averaged AUC of 0.844 while the radiology residents and pulmonologists yielded a mean AUC of 0.804, both performances were significantly different from the computer model (p < 0.001 and p = 0.002, respectively).

**Fig 1:**
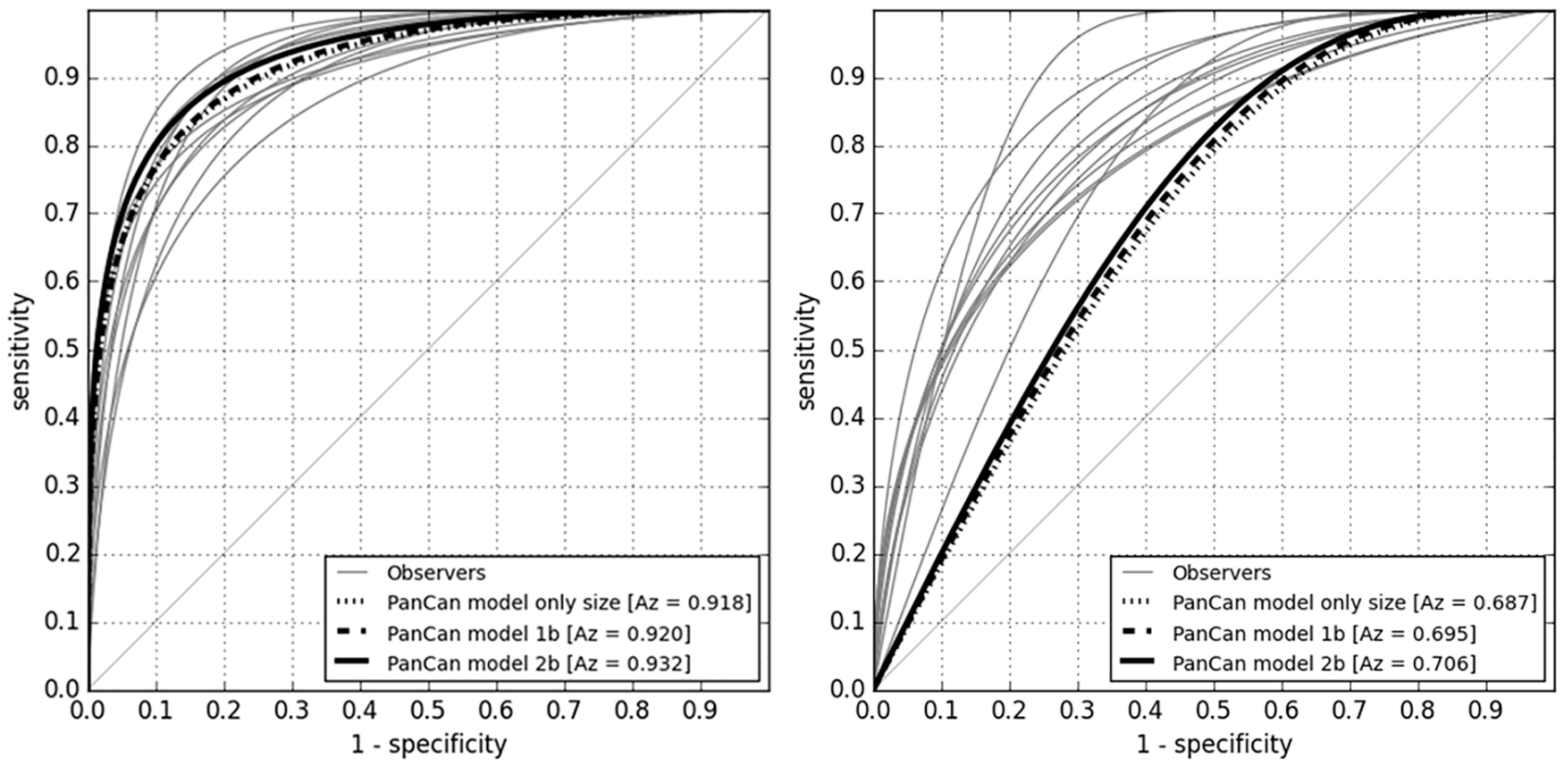
Performance of observers and PanCan model. ROC curves of the observers 1–11, the PanCan model 2b, the PanCan model 1b, and only nodule size as predictor for (A) discriminating randomly selected benign nodules from malignant nodules on the left, and (B) on the right discriminating size-matched benign nodules from malignant nodules. Note that in Fig 1A the PanCan model outperforms human observers at a specificity > 80%, while in Fig 1B all human observers perform better than the PanCan model.

**Fig 2:**
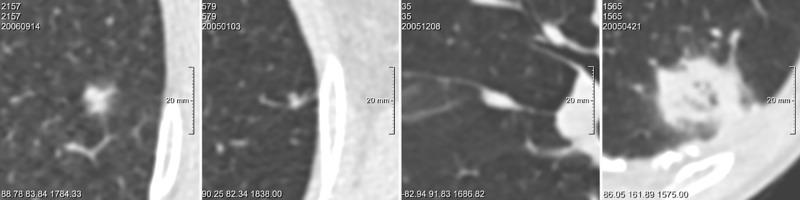
Observer agreement for malignancy probability score. Examples of nodules for which observers and the PanCan model uniformly scored a high or low malignancy probability. For the observers, a threshold of < 25% averaged over all observers was considered a 'low risk' score, and a threshold of > 60% was considered a 'high risk' score. For the PanCan model, a threshold of < 6% was considered as a 'low risk' score and a threshold of > 30% was considered as a 'high risk' score. Nodules are displayed in the axial plane. From left to right: A) Part-solid *malignant* nodule, 13 mm, observers scored 65%, PanCan 31.9% corresponding to a uniformly true positive score; B) Solid *malignant* nodule, 4 mm, observers scored 9.5%, PanCan 0.3%; corresponding to a uniformly false negative score; C) Solid *benign* perifissural nodule, 11 mm, observers scored 1.9%, PanCan 0%, corresponding to a uniformly true negative score; D) Part-solid *benign* nodule, 27 mm, observers scored 60%, PanCan 30.7%, corresponding to a uniformly false positive score.

**Fig 3:**
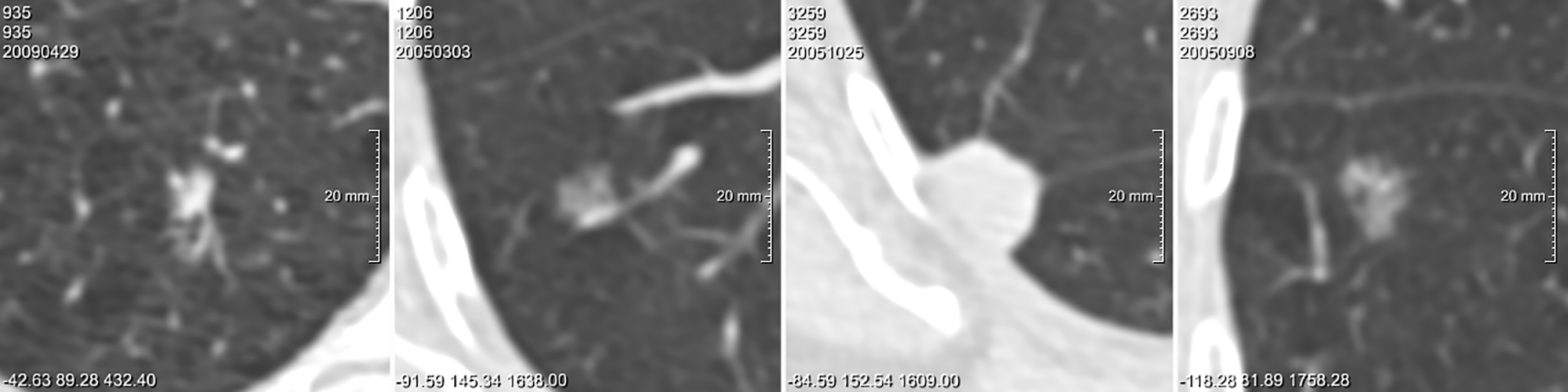
Observer disagreement for malignancy probability score. Examples of nodules for which the PanCan model and the observers showed conflicting malignancy probability scores. For the observers, a threshold of < 25% averaged over all observers was considered a 'low' score, and a threshold of > 60% was considered a 'high' score. For the PanCan model, a threshold of < 6% was considered a 'low' score and a threshold of > 30% was considered a 'high' score. Nodules are displayed in axial plane. From left to right: A) Solid *malignant* nodule, 15 mm, observers scored 24%, PanCan 35%; B) Pure ground-glass *malignant* nodule, 9 mm, observers scored 58%, PanCan 4%; C) Solid *benign* nodule, 16.5 mm, observers scored 14%, PanCan 37%; D) Part-solid *benign* nodule, 13 mm, observers scored 65%, PanCan 14%.

The parsimonious model achieved an AUC of 0.920 for the randomly selected benign and 0.695 for the size-matched benign nodules. When nodule size was considered as the only input parameter for the model, the AUC yielded 0.918 for randomly selected benign and 0.687 for size-matched benign nodules, respectively. Table 2 displays the AUC of each observer, the AUC averaged over all observers and the AUC of the computer model for the two discrimination tasks. Fig 1 shows the corresponding ROC curves. Fig 2 shows examples of nodules classified uniformly by all observers and the computer model, while Fig 3 depicts examples of nodules for which discrepant risk estimations occurred between the computer model and observers.

**Table 2:** Performance of observers and the various computer models (PanCan 1b and 2b) for the discrimination between malignant and benign nodules for random and size-matched benign nodules versus malignant nodules.

Readers	AUC* random nodules	P-value	AUC size-matched nodules	P-value
**Observer 1**	0.902	0.285	0.797	**0.027**
**Observer 2**	0.909	0.346	0.820	**0.002**
**Observer 3**	0.936	0.793	0.877	**<0.001**
**Observer 4**	0.928	0.903	0.881	**0.004**
**Observer 5**	0.930	0.910	0.800	**0.009**
**Observer 6**	0.950	0.274	0.771	0.066
**Observer 7**	0.881	**0.049**	0.846	**<0.001**
**Observer 8**	0.860	**0.004**	0.783	**0.049**
**Observer 9**	0.901	0.073	0.786	**0.023**
**Observer 10**	0.896	0.173	0.813	**0.005**
**Observer 11**	0.915	0.499	0.830	**0.004**
**Average all**	0.910	0.184	0.819	**<0.001**
**Board certified radiologists (1–4)**	0.919	0.366	0.844	**<0.001**
**Radiology residents and pulmonologists (5–11)**	0.905	0.117	0.804	**0.002**
**PanCan model 2b**	0.932		0.706	
**PanCan model 1b**	0.920		0.695	
**PanCan, size only**	0.918		0.687	

* AUC randomly selected nodules: area under the receiver-operating-characteristics curve for discriminating malignant from randomly selected benign nodules. AUC size-matched nodules: area under the receiver-operating-characteristics curve for discriminating malignant from size-matched benign nodules. P-values refer to comparison with the PanCan model 2b. A significant difference is defined at p-values < 0.05 and significant differences are indicated in bold.

### Malignancy probability scores

In the full computer model, the median malignancy probability score was 18.5% (range 0.3%–85.7%) for malignant nodules, 4.6% (range 0%–67.8%) for size-matched benign nodules, and 0.4% (range 0%–25.9%) for randomly selected benign nodules. For the parsimonious model the corresponding numbers were 13.6% (range 0.2%–86.7%), 4.4% (range 0%–62.1%), and 0.3% (range 0%–18.1%).

**Fig 4:**
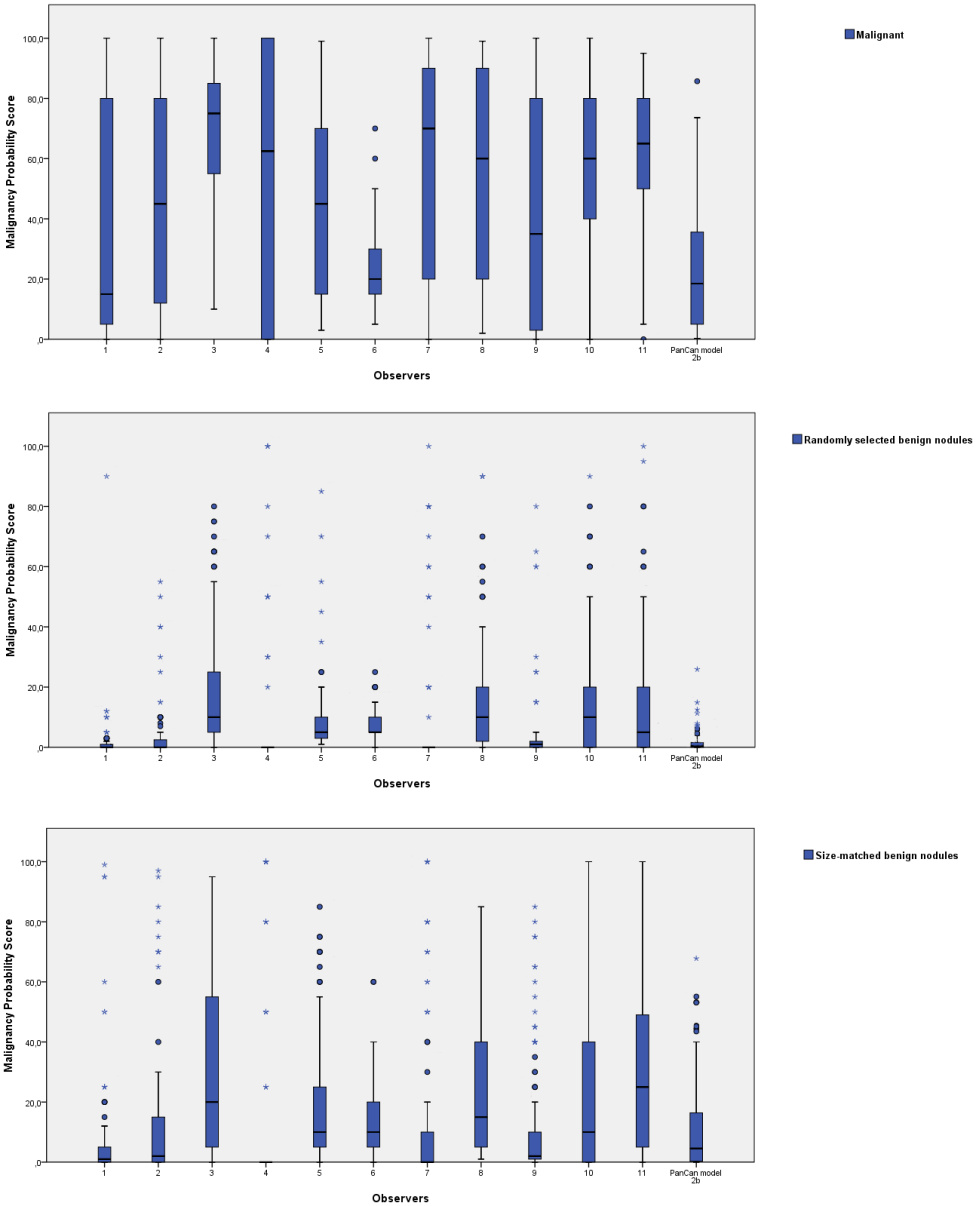
Malignancy probability score distribution. The distribution of malignancy probability scores of the observers and the PanCan model are visualized in these box plots for the malignant nodules in the upper box plot (A), randomly selected benign nodules in the middle box plot (B), and size-matched benign nodules in the lower box plot (C). Observers and the PanCan model use a different distribution of the scale (0–100). Note the large variability between observers with respect to mean and range of risk probability estimates.

The median probability scores determined by the observers were 50% (range 15%–75%) for malignant nodules, 5% (range 0%–25%) for size-matched and 2% (range 0%–10%) for randomly selected benign nodules. The distribution of malignancy probability scores for the three nodule groups per observer and the computer model are depicted in Fig 4.

### Morphological characteristics

Seven observers determined the morphological characteristics of nodules. Table 3 displays the morphological features scored by the observers. Morphological features that were statistically significantly predictive of malignancy in the majority of the observers were spiculation (p < 0.001), distortion of surrounding lung parenchyma architecture (p < 0.001) and retraction of pleura or fissure (p = 0.002). A well-defined border was statistically significantly predictive of a benign nodule (p < 0.001). Spiculation is the only feature that is included in the PanCan model 1b and 2b.

**Table 3:** Differences in morphological features between malignant and size-matched benign nodules.

Morphology characteristics	Malignant nodules n = 62 *	Benign size-matched nodules n = 118 *	P-value
Single bubble	2 (3%)	1 (1%)	0.236
Multiple bubbles	6 (10%)	3 (3%)	0.037
Airbronchogram	5 (8%)	4 (3%)	0.171
Bulla with thickened wall	4 (6%)	0 (0%)	0.005
Spiculation	20 (32%)	4 (3%)	**<0.001**
Lobulation	7 (11%)	3 (3%)	0.015
Ill-defined border	19 (31%)	25 (21%)	0.161
Well-defined border	10 (16%)	65 (55%)	**<0.001**
Demarcation by interlobular septum	0 (0%)	1 (1%)	0.467
Attachment to vessel	11 (18%)	6 (5%)	0.006
Attachment to pleura	23 (37%)	45 (38%)	0.891
Attachment to fissure	10 (16%)	13 (11%)	0.329
Retraction of pleural or fissure	12 (19%)	6 (5%)	**0.002**
Distortion of surrounding lung architecture	14 (23%)	7 (6%)	**<0.001**

* Percentages are in parentheses. Only a positive score occurred when ≥ four of the seven observers considered “feature being present”. A p-value < 0.004 was considered to indicate significance of difference. Significant differences are indicated in bold.

**Fig 5:**
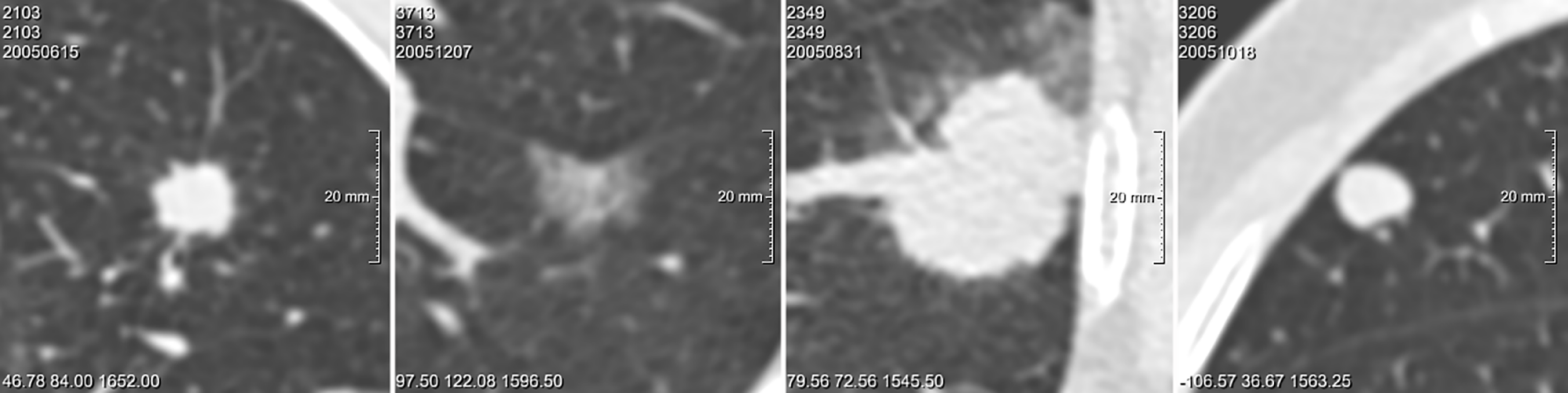
Examples of nodules with morphological characteristics uniformly scored by six or seven observers. Every nodule is displayed in axial plane. Images show a field of view of 60 x 60 mm, in which the nodule is centered. From left to right: A) Solid malignant nodule, 13 mm, with spiculation; B) Part-solid malignant nodule, 17 mm, with retraction of a fissure; C) Solid malignant nodule, 30 mm, with distortion of surrounding architecture; D) Solid benign nodule, 11 mm, with a well-defined border.

Fig 5 shows typical examples of four morphological characteristics that showed significant differences.

## Discussion

Given the large discrepancy between the prevalence of benign and eventually malignant nodules in a screening population, robust methods for risk prediction are mandatory in a screening program for accurate discrimination of high-risk lesions that require additional work-up and low-risk lesions requiring none or less intense follow-up. Given the large number of nodules and scans to be evaluated and the influence of observer variability leading to loss of standardization and even potential oversights, application of a computer model for risk prediction appears a very attractive alternative. The PanCan model published in 2013 was the first mathematical model for predicting the risk of malignancy in screen-detected nodules that was derived from an actual screening population (the Pan-Canadian Early Detection of Lung Cancer Study). Subsequently, two external validations confirmed the high performance of the PanCan model in screening populations [16, 18, 24] one using the BCCA population (AUC > 0.900) and one the DLCST screening trial (AUC 0.834). One study comparing four prediction models for the assessment of clinically detected lung nodules found a performance for the PanCan model of AUC = 0.902, compared to 0.735 for the Veterans Association model and 0.895 for the Mayo model. The only model achieving a higher AUC was the Herder model with 0.924 when including information of FDG PET-CT [24].

In a previous study of our research group we had compared the PanCan model to other management strategies such as National Comprehensive Cancer Network (NCCN) or Lung-RADS for risk estimation of screen-detected nodules and also found a superiority of PanCan to the other regimen, with an AUC of 0.874 compared to 0.813 (Lung-RADS, p = 0.003) and to 0.836 (NCCN, p = 0.010) [25].

We therefore were particularly interested in comparing the performance of the PanCan computer model to human observers, which has never been done before.

To optimize between observer time and acquisition of statistically meaningful data, we used a case-controlled study set-up. Therefore, our study design has a different prevalence of malignant nodules (~33%) compared to, for example, the NLST (< 3%). Although there is little research on the effect of prevalence expectation on the performance of observers, the available research suggests that the effect is minimal [26]. Additionally, this study design is not suited to be used as external validation of the performance of the PanCan model for a screening cohort as has been done previously using the BCCN and the Danish lung cancer screening dataset.

In fact, our results confirm the high performance of the PanCan model in a representative subset of the DLCST baseline population for discriminating malignant from randomly selected benign nodules. The PanCan model was slightly, yet not significantly superior to the average human observer performance (0.932 versus 0.910, p = 0.184). Based on this result it can be concluded that the PanCan model represents a very valuable tool that is equally effective for discriminating high-risk from low-risk screen-detected nodules as radiologists with varying experience.

Our results underline the importance of size for predicting malignancy risk [27]. In fact, using only size as the single indicator provided comparable AUCs as using the full PanCan model, as illustrated in Fig 1. For the task of differentiating malignant from benign nodules of similar size, the observers as well as the PanCan model achieved a substantially lower performance than for the task of discriminating malignant from randomly selected benign nodules. This can be explained by the fact that increasing size alone represents a very strong risk factor. However, by taking visually accessible morphological aspects other than size and spiculation into account, the human observers achieved a superior performance to the PanCan model for this dataset (AUC 0.819 and 0.706, respectively, p < 0.001). The morphological characteristics that were rated with different frequency in malignant and benign lesions were related to border characteristics (spiculation and well-defined border) and interference of the nodule with the perinodular lung architecture (retraction of the pleura or fissure, distortion of the surrounding architecture). While it can be assumed that these features did have an impact on the observers’ judgment, the findings that make an observer rate a lesion as suspicious are most likely more complex. Similar results were reported by Chung et al. [28] who reported a significant increase of correctly updated subsolid lesions to Lung-RADS category 4B, which represents the most suspicious and highest risk category based on visual analysis. Similar to our results, inter-reader variability was substantial and observer data did not allow to pinpoint a single group of morphological features being significantly predictive.

Although visual analysis of morphology apparently provides very useful information with substantial discriminative power, none of the features were completely discriminative, and there seems to be substantial observer variability as indicated by the substantial inter-reader variability for the malignancy probability scores. The latter was demonstrated by one observer (number 6) to an extreme: while achieving the best performance for discriminating malignancies versus randomly selected benign nodules, the observer demonstrated the poorest performance for discriminating malignancies from size-matched benign nodules. In general, more experienced observers achieved a higher performance than less experienced observers (AUC 0.919 vs. 0.905 for random subset; 0.844 vs. 0.804 for size-matched subset).

Another way to further improve the model's performance could be to include nodule growth between scans obtained at different time points. Several studies have shown [29–30] that lesion growth over time is the most important and powerful predictor of nodule malignancy. Recently Horeweg et al. demonstrated that volume doubling time (VDT) can be used as an additional feature to individualize management of intermediate-sized nodules (5–10 mm). Using the database of the Dutch-Belgian lung cancer screening trial, the authors reported a malignancy risk of 0.8% for a volume doubling time (VDT) of ≥ 600 days, a risk of 4% for a VDT of 400–600 days and a risk of 9.9% for a VDT of ≤ 400 days [31].

It has to be noted that the observer probability scores on a scale from 0 to 100 cannot be directly transferred to the probability estimates of the PanCan model. Some observers used only the lower end of the range between 0 and 100, while others used a more wide-spread distribution. Following the PanCan model output thresholds as described by Tammemagi et al. [32], a score of < 6% represents a low-risk lesion triggering annual repeat screening, an intermediate score between 6% and < 30% triggers a rescreen in 3 months and a score of ≥ 30% indicates a high-risk lesion requiring more intense and possibly invasive diagnostic work-up. The PanCan model rarely achieves scores beyond 50%. There is an inherent discrepancy in the process of risk estimation of a logical but rigid mathematical model and the intuitive but variable visual analysis of human observers. Whereas a direct comparison of the scores does not lead to meaningful conclusions, the ROC statistical analysis we used sufficiently considered the relative distribution of the scores and therefore allowed for comparing the performances of observers and the PanCan model.

Our study has limitations. In the size-matched experiment, we tried to match the benign nodules in size to the malignant nodules as close as possible but perfect matching was not possible. As a result, the mean diameter of the malignant lesions was 3 mm larger than the mean of the benign nodules. However, while introducing a bias, it affected both observers and mathematical model in the same direction.

Another limitation refers to the different inclusion criteria of the PanCan model and the DLCST: perifissural and calcified nodules were specifically excluded in the PanCan model because of most likely being benign. In the DLCST trial, however, calcified and perifissural nodules were included as solid nodules. Both, calcified and perifissual nodules in the group of randomly selected and size-matched benign nodules were therefore assigned a score of 0% in our study, to account for this design difference.

Furthermore, our dataset comprised a relatively small number of lung cancers (62 in total), although we included all participants with malignant nodules annotated in the DLCST.

Finally, inter-observer variability was not further quantified, however it's well reflected in the variable AUC of the observers and the large variability of risk scores (Fig 4), as can be expected with a large number of observers.

In conclusion, the PanCan risk prediction model and human observers perform equally well for differentiating malignant from randomly selected benign screen-detected pulmonary nodules, underlining the large potential of computer based risk estimation to trigger nodule management in population based screening studies. Human observers, however, significantly outperform the PanCan model for differentiating malignant from size-matched screen-detected benign nodules suggesting that integration of additional morphological characteristics, such as pleural retraction and perinodular lung parenchyma distortion, used by the human observers is very likely to lead to further improvement of computer based risk prediction models.

## Abbreviations

ACR: American College of Radiologists
AUC: area under the receiver-operator-characteristics curve
BCCA: British Columbia Cancer Agency
CT: computed tomography
DLCST: Danish Lung Cancer Screening Trial
NLST: National Lung Screening Trial
ROC: receiver operating characteristic curve
USPSTF: United States Preventative Services Task Force
